# Concurrent Occurrence of Neuropsychiatric Systemic Lupus Erythematosus and Sarcoidosis: A Case Report

**DOI:** 10.7759/cureus.46464

**Published:** 2023-10-04

**Authors:** Manish Kharel, Bibek Parajuli, Sunil Timilsina, Suchit Thapa Chhetri, Bishal Kunwor

**Affiliations:** 1 Medicine and Surgery, Jahurul Islam Medical College, Dhaka, BGD; 2 Internal Medicine, Gandaki Medical College, Pokhara, NPL; 3 General Practice and Emergency Medicine, Shree Birendra Hospital, Kathmandu, NPL; 4 Medical School, Nepalese Army Institute of Health Sciences, Kathmandu, NPL

**Keywords:** autoimmune disorder, genetic predisposition, autoantibodies, sarcoidosis, systemic lupus erythematosus

## Abstract

Systemic lupus erythematosus (SLE) and sarcoidosis are both complex autoimmune disorders with varying clinical manifestations. The incidence of SLE is as low as 4.91 per 100,000 population, and that of sarcoidosis is 0.85 per 100,000 population. The prevalence of neuropsychiatric systemic lupus erythematosus (NPSLE) ranges from 17.6% to 44.5%. The concurrent occurrence of NPSLE and sarcoidosis, although rare, presents diagnostic and management challenges. The clinical picture resulting from the coexistence of NPSLE and sarcoidosis, which share a common immunological picture, is not well defined. This case report discusses a patient with coexisting NPSLE and sarcoidosis, highlighting the intricate interplay between these conditions.

## Introduction

Systemic lupus erythematosus (SLE) is a rare, chronic multisystemic autoimmune illness that can range from mild to potentially life-threatening [1]. The incidence of SLE is as low as 4.91 per 100,000 people [1]. Fatigue, joint pain, fever, myalgia, skin rashes, photosensitivity, shortness of breath, and alopecia are the most common presenting symptoms of SLE [2]. Neuropsychiatric manifestations can be seen in 39% to 50% of SLE patients [3]. The prevalence of neuropsychiatric systemic lupus erythematosus (NPSLE) ranged from 17.6% to 44.5% in retrospective and prospective studies in a meta-analysis that included 5,057 SLE patients, respectively [2]. The reported prevalence and incidence were 4.3% and 7.8%, respectively, after removing the minor symptoms [2]. Some of the common neuropsychiatric manifestations in SLE patients include headache, cognitive dysfunction, mood disorders, seizure disorders, cerebrovascular disease, and anxiety disorder [4].

Sarcoidosis is a multisystemic disorder of unknown cause, commonly affecting young and middle-aged adults with symptoms involving various organs and diagnosed by histological evidence of noncaseating epithelioid cell granulomas and an aggravated T helper cell type 1 (Th1) immune response [5]. The incidence of sarcoidosis is very low, ranging from 0.81 to 0.85 per 100,000 people [6].

The clinical presentation of individuals with concurrent NPSLE and sarcoidosis is not well defined and can be challenging to differentiate due to their shared immunological and neurological features. Concurrent findings of neuropsychiatric SLE and sarcoidosis have been rarely reported in the literature [7].

## Case presentation

A 45-year-old African-American man, previously diagnosed with sarcoidosis 10 years ago, presented to the emergency room with complaints of double vision on the lateral gaze and experiencing numbness, tingling, and proximal muscle weakness while lifting things against gravity and climbing stairs. Additionally, he had a history of mild chronic obstructive pulmonary disease and was using an albuterol inhaler as part of his prescribed treatment plan, taking one to two inhalations (puffs) as needed every four to six hours for symptom relief. Over the past six months, he had been troubled by a persistent dry cough.

During the physical examination, it was observed that the individual had a slender physique and exhibited normal vital signs. Both of his eyes displayed limitations in abduction, indicating the presence of bilateral sixth cranial nerve neuropathies. He scored a 3/5 for both proximal muscular atrophy and proximal weakness in the upper and lower extremities. The patient scored a 5/5 on distal muscle strength. The patient reported feeling a slight discomfort in his muscles, described as occasional cramping sensations that were transient and occurred intermittently. Reflexes on the left side were entirely absent, and the patient exhibited a notable decrease in sensation in the distal legs, encompassing reduced proprioception, light touch, and vibration sense. An abdominal examination was benign. The laboratory test results are listed in Table 1.

**Table 1:** The patient's laboratory test results at the time of admission

Test	Result	Reference Range
Hemoglobin	10 g/dL	13.5 - 17.5 g/dL
Aspartate Aminotransferase	32 U/L	0-40 U/L
Total Bilirubin	1.44 mg/dl	0.2-1.2 mg/dL
Calcium	9.02 mg/dL	8.5-10.5 mg/dL
Creatinine	0.76 mg/dL	0.6-1.3 mg/dL
Vitamin B12	673 pg/mL	200-900 pg/mL
Folate	11 ng/mL	3-17 ng/mL
Creatinine Protein Kinase	Normal	Normal range varies by age and gender
Aldolase	3 U/L	1-8 U/L
Rapid Plasma Reagin	Normal	Non-reactive
Thyroid Stimulating Hormone	2.6 mIU/L	0.4-4.0 mIU/L
C-reactive Protein (CRP)	3 mg/L	0-10 mg/L
Erythrocyte Sedimentation Rate (ESR)	7.8 mm/hr	0-15 mm/hr
Proteinuria	3+	Absent or trace

**Figure 1:**
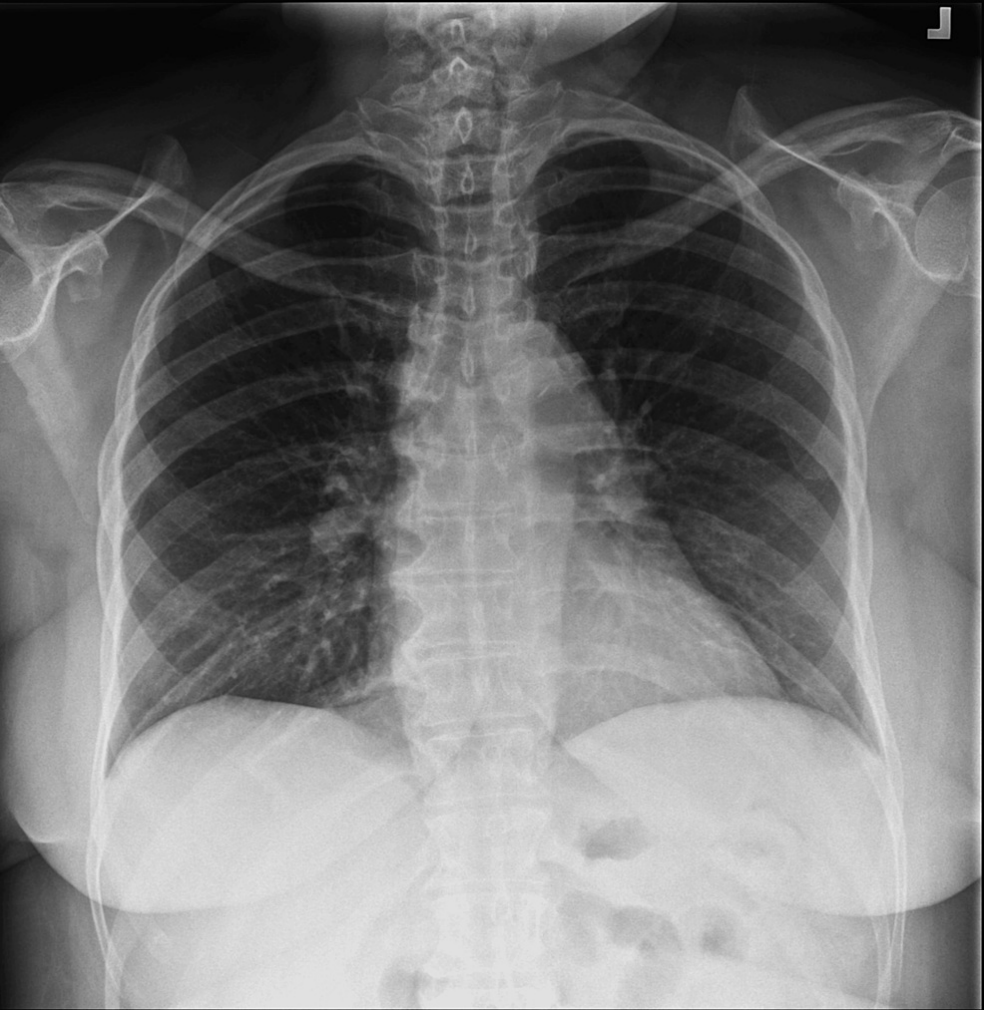
A chest X-ray revealed clear lungs with sharp costophrenic angles, but mild apical pleural thickening was noted.

The results of a head CT scan were normal. The chest X-ray revealed mild apical pleural thickening (Figure 1).

**Figure 2:**
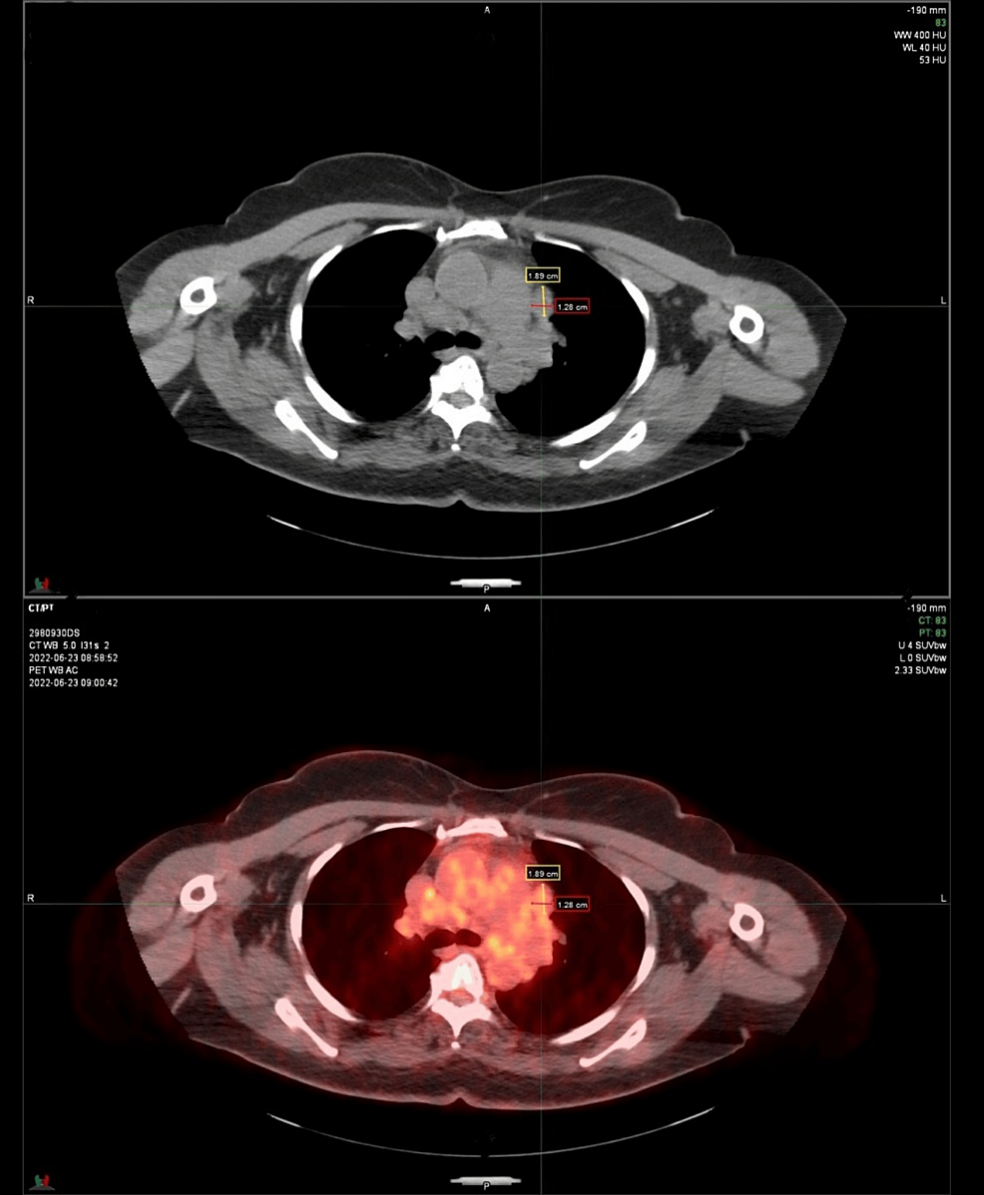
The PET-CT scan of the chest revealed a 2.8 cm mildly hypermetabolic nodular opacity in the left upper lung lobe and a 0.6 cm nodular opacity near the right major fissure with mildly hypermetabolic mediastinal and bilateral hilar lymph nodes and no enlarged lymphadenopathy in the neck, chest, abdomen, and pelvic areas.

A positron emission tomography-computed tomography (PET-CT) scan of the chest revealed several calcified nodules that were slightly enlarged (Figure 2).

A broad, primarily axonal sensory-motor length-dependent polyneuropathy and myopathy with some evidence of a mononeuritis multiplex undergoing generalized transformation were revealed by electromyography (EMG) and a nerve conduction study (NCS). After reviewing the patient's medical history, a sarcoidosis exacerbation was identified.

The patient was treated with a dosage of 40 mg of prednisone for presumed sarcoidosis exacerbation, but no response was observed even after receiving five doses of the steroid. For a paraneoplastic etiology, a workup was conducted. The CT scan revealed a normal abdomen. The results of the colonoscopy and prostate-specific antigen (PSA) test were both normal, indicating a healthy colon and prostate. An inflammatory infiltrate with enhanced plasma cells was found in the lung infiltrate after a fine needle aspiration biopsy in the left upper region; however, there were no granulomas, and all cultures were negative. The laboratory findings in the rheumatology evaluation are listed in Table 2.

**Table 2:** Laboratory findings in the rheumatology evaluation

Test / Finding	Result
Antinuclear Antibody (ANA)	>1:800 (Positive)
Anti-double Stranded DNA Antibodies	Substantially positive in other profiles
Beta-2 Glycoprotein-1 Antibodies	Negative
Lupus Anticoagulant	Negative
Antineutrophil Cytoplasmic Antibodies	Negative
Anticardiolipin Antibodies	Negative
Anti-Hu Antibodies	Negative
Anti-Ri Antibodies	Negative
Anti-Yo Antibodies	Negative
Muscle Specific Kinase (MuSK) Antibodies	Negative
Anti-voltage-Gated Calcium Channel Antibodies	Negative
Anti-acetylcholine Receptor Antibodies	Negative
Gq1b Antibodies	Negative
Angiotensin-Converting Enzyme	Negative
Anti-Lyme Antibodies	Negative
24-Hour Urine Protein	2 grams
Direct/Indirect Coombs	Positive
Serum Haptoglobin Levels	Reduced

The patient was identified as having NPSLE with concomitant sarcoidosis based on the presence of autoantibodies, Coombs-positive hemolytic anemia, proteinuria, symmetrical progressive sensorimotor polyneuropathy with active sarcoidosis in the lung, cranial neuropathies, and a history of psychosis.

The patient began receiving intravenous (IV) cyclophosphamide at a dosage of 1000 mg/m^2 about three weeks after his initial presentation, and within four weeks, both his motor strength and extraocular movement had significantly improved. He has currently had six cyclophosphamide infusions, is off prednisone, and is responding well to methotrexate. His anemia and diplopia have completely disappeared. The patient is doing well on follow-ups.

## Discussion

The clinical picture resulting from the coexistence of NPSLE and sarcoidosis, which share a common immunological picture, is not well defined. Both SLE and sarcoidosis exhibit cellular and humoral immunological abnormalities, such as immune system hyperreactivity, hypergammaglobulinemia, impaired cell-mediated immune function, and loss of self-antigen tolerance [5]. In our case, the potential differential diagnoses considered for the patient's condition included sarcoidosis exacerbation due to the patient's medical history, NPSLE considering cranial neuropathies and autoantibodies, chronic inflammatory demyelinating polyneuropathy (CIDP) based on progressive muscle weakness and sensory deficits, mononeuritis multiplex associated with systemic conditions, and paraneoplastic syndrome as a consideration to rule out malignancies with neurological symptoms.

Similar clinical signs, such as polyarthritis, fever, or peripheral lymphadenopathy, can be present in both sarcoidosis and SLE. Approximately 0.5%-1% of neuropsychiatric manifestations of SLE are cranial palsies, an uncommon central nervous system expression [8]. The eighth, oculomotor (third, fourth, and sixth) nerves are most frequently affected by cranial neuropathies, while the fifth and seventh nerves are less frequently affected [8]. Ten percent of sarcoidosis patients had central nervous system (CNS) involvement, with cranial involvement occurring in 50% to 75% of cases, most frequently affecting the facial nerve [9]. In our case, there was sixth cranial nerve neuropathy on both sides.

Patients with SLE who present with interstitial lung disease and bilateral hilar lymphadenopathy should be evaluated for the presence of sarcoidosis since these are rare in SLE alone [10]. The most frequent form of sarcoidosis presentation is pulmonary manifestation, which often has a good prognosis. Begum et al. detected three cases (1%) of concomitant sarcoidosis and SLE in a group of 300 patients with SLE [11].

Since there are elusive signs and symptoms for diagnosis, NPSLE management is difficult. The management of NPSLE is centered on symptomatic treatment, such as antiepileptics for seizures, anxiolytics or antipsychotics for psychiatric symptoms, antihypertensive medicines for hypertension, and correction of metabolic derangements [12]. Immunosuppressants like corticosteroids should be started in patients with immune-mediated injury or generalized lupus activity, either alone or in conjunction with other immunosuppressive therapies. A better knowledge of the immunological mechanisms underlying active SLE is leading to the development of new treatments and targets. However, NPSLE has no possible adversary. For people with the active disease of sarcoidosis who exhibit symptoms, the first line of treatment advised is systemic corticosteroids [13].

Sarcoidosis and SLE share a genetic predisposition. Lupus is associated with the alleles HLA-B8, HLA-A1-B8-DR3, and HLA-DR2 [14,15]. Despite the paucity of genetic investigations into sarcoidosis, one risk allele, HLA-B8-DR3, has been identified [16]. To fully comprehend these disorders, more work is required on genetics and etiology.

## Conclusions

It is important to take into account the concurrence of sarcoidosis and SLE. Rheumatologists may have difficulty diagnosing NPSLE due to the lack of sensitive and precise laboratory serum or cerebrospinal (CSF) fluid biomarkers, nuclear and radiological imaging, and other formal criteria for diagnosing and directing management decisions in NPSLE. Therefore, further research should be conducted to investigate the coexistence of sarcoidosis and SLE, aiming to enhance our comprehension of this association and facilitate improved co-management of both conditions.
